# Supplementary material for: Long term intrinsic cycling in human life course antibody responses to influenza A(H3N2): an observational and modeling study
Source: eLife. 2022 Dec 2;11:e81457. doi: 10.7554/eLife.81457 (PMC9757834; doi:10.7554/eLife.81457)
Supplement: Figure 1—source data 1. [file elife-81457-fig1-data1.docx]

## Figure 1—source data 1. Variance (%) explained by low frequencies and peak frequencies for Fourier spectra of individual residuals.

|  | **All participants** | **Participants born before 1968** |
| --- | --- | --- |
| **Low frequency (0.025 to 0.050)** |  |  |
| Baseline | 22.1 (11.1, 35.4) | 19.8 (10.0, 32.2) |
| Follow-up | 21.9 (10.3, 35.5) | 18.7 (9.1, 32.2) |
| **Peak frequency** |  |  |
| Baseline | 36.4 (29.3, 45.1) | 33.9 (28.0, 42.2) |
| Follow-up | 36.8 (30.1, 45.9) | 34.7 (28.6, 42.2) |
