# Supplementary material for: Long term intrinsic cycling in human life course antibody responses to influenza A(H3N2): an observational and modeling study
Source: eLife. 2022 Dec 2;11:e81457. doi: 10.7554/eLife.81457 (PMC9757834; doi:10.7554/eLife.81457)
Supplement: Figure 3—source data 1. [file elife-81457-fig3-data1.docx]

## Figure 3— source data 1. Parameters used in the simulations.

| **Parameters** | **Definitions** | **Value** | **Note** |
| --- | --- | --- | --- |
| Individual Immune responses | | | |
| $\mu_{l}$ | Mean log titers of long-term (i.e., broad range) boosting to an infected strain. | 2.02 | Ref ((*4*)) |
| $\mu_{s}$ | Mean log titers of short-term (i.e., narrow range) boosting to an infected strain. | 2.69 | Ref ((*4*)) |
| $\sigma_{l}$ | Duration in years to determine the decrease of cross-reactions for long-term (i.e., broad range) boosting. | 1/0.130 | Ref ((*4*)) |
| $\sigma_{s}$ | Duration in years to determine the decrease of cross-reactions for short-term (i.e., narrow range) boosting. | 1/0.031 | Ref ((*4*)) |
| $\omega$ | Duration in years for the decay of short-term (i.e., narrow range) boosting. | 1/0.79 | Ref ((*4*)) |
| $\tau$ | Parameter to determine the antigenic seniority. | 0 | Assumption |
| $\rho$ | Rate of changes in antigenic distance per year. | 0.778 | Ref ((*3*)). |
| Immunity-dependent protection | | | |
| $\beta$ | Scale parameter of the titer-dependent protection. | 2.1 | Ref ((*37*)) |
| $\mu_{50}$ | Log titer with 50% probability of protection from infection. | 3 | Ref ((*37*)) |
| Population-level circulation | | | |
| $\lambda_{t}$ | A(H3N2) activity in year t. | a) a constant of 0.2;  b) random, Uniform (0, 0.2); c) cyclic with a 5-year periodicity and maximum activity of 0.2 | Assumption.  Equation 11 |
