# Supplementary material for: Long term intrinsic cycling in human life course antibody responses to influenza A(H3N2): an observational and modeling study
Source: eLife. 2022 Dec 2;11:e81457. doi: 10.7554/eLife.81457 (PMC9757834; doi:10.7554/eLife.81457)
Supplement: Figure 3—source data 2. [file elife-81457-fig3-data2.docx]

## Figure 3—source data 2. Mechanisms examined in the simulations.

| **Mechanism** | **Definition** | **Parameters** | **Note** |
| --- | --- | --- | --- |
| **Infection risk determinant** | | | |
| Immunity-dependent protection | Lower risk of infection with higher HI titer to a circulating A(H3N2) strain. | $\lambda_{t}$, $\beta$, $\mu_{50}$ | Equation 10 & 11 |
| Population-level circulation | Constant, random, or periodic fluctuation in population-level circulation of the viruses. | $\lambda_{t}$ | Equation 11 |
| **Antibody responses** |  |  |  |
| Broad cross-reaction | Interaction of antibody responses to strains that were isolated distantly. | $\mu_{l}$, $\mu_{s}$, $\sigma_{l}$, $\sigma_{s}$, $\rho$ | Equation 4 |
| Narrow cross-reaction | Interaction of antibody responses to strains that are antigenically similar. | $\mu_{l}$, $\mu_{s}$, $\sigma_{l}$, $\sigma_{s}$, $\rho$ | Equation 5 |
